# Perinatal Manganese Exposure and Hydroxyl Radical Formation in Rat Brain

**DOI:** 10.1007/s12640-014-9474-z

**Published:** 2014-05-09

**Authors:** Michał Bałasz, Ryszard Szkilnik, Ryszard Brus, Jolanta Malinowska-Borowska, Sławomir Kasperczyk, Damian Nowak, Richard M. Kostrzewa, Przemysław Nowak

**Affiliations:** 1 Department of Toxicology and Occupational Health Protection, Public Health Faculty, Medical University of Silesia, Medykow 18, 40-752 Katowice Ligota, Poland; 2 Department of Nurse, High School of Strategic Planning, Koscielna 6, 41-303 Dabrowa Gornicza, Poland; 3 Department of Basic Medical Sciences, Medical University of Silesia, Piekarska 18, 41-902 Bytom, Poland; 4 Department of Biochemistry, Medical University of Silesia, 19 Jordana Str, 41-808 Zabrze, Poland; 5 Department of Biomedical Sciences, Quillen College of Medicine East Tennessee State University, P.O. Box 70577, Johnson City, TN 37614 USA

**Keywords:** Manganese, Ontogenetic, 6-hydroxydopamine, Brain, Hydroxyl radicals, Rats

## Abstract

The present study was designed to investigate the role of pre- and postnatal manganese (Mn) exposure on hydroxyl radical (HO^•^) formation in the brains of dopamine (DA) partially denervated rats (Parkinsonian rats). Wistar rats were given tap water containing 10,000 ppm manganese chloride during the duration of pregnancy and until the time of weaning. Control rat dams consumed tap water without added Mn. Three days after birth, rats of both groups were treated with 6-hydroxydopamine at one of three doses (15, 30, or 67 µg, intraventricular on each side), or saline vehicle. We found that Mn content in the brain, kidney, liver, and bone was significantly elevated in dams exposed to Mn during pregnancy. In neonates, the major organs that accumulated Mn were the femoral bone and liver. However, Mn was not elevated in tissues in adulthood. To determine the possible effect on generation of the reactive species, HO^•^ in Mn-induced neurotoxicity, we analyzed the contents of 2.3- and 2.5-dihydroxybenzoic acid (spin trap products of salicylate; HO^•^ being an index of in vivo HO^•^ generation), as well as antioxidant enzyme activities of superoxide dismutase (SOD) isoenzymes and glutathione S-transferase (GST). 6-OHDA-depletion of DA produced enhanced HO^•^ formation in the brain tissue of newborn and adulthood rats that had been exposed to Mn, and the latter effect did not depend on the extent of DA denervation. Additionally, the extraneuronal, microdialysate, content of HO^•^ in neostriatum was likewise elevated in 6-OHDA-lesioned rats. Interestingly, there was no difference in extraneuronal HO^•^ formation in the neostriatum of Mn-exposed versus control rats. In summary, findings in this study indicate that Mn crosses the placenta but in contrast to other heavy metals, Mn is not deposited long term in tissues. Also, damage to the dopaminergic system acts as a “trigger mechanism,” initiating a cascade of adverse events leading to a protracted increase in HO^•^ generation, and the effects of Mn and 6-OHDA are compounded. Moreover, HO^•^ generation parallels the suppression of SOD isoenzymes and GST in the brains of rats lesioned with 6-OHDA and/or intoxicated with Mn—the most prominent impairments being in frontal cortex, striatum, and brain stem. In conclusion, ontogenetic Mn exposure, resulting in reactive oxygen species, HO^•^ formation, represents a risk factor for dopaminergic neurotoxicity and development of neurodegenerative disorders.

## Introduction

Manganese (Mn) is an enzymatic cofactor that plays an important role in a number of physiologic processes. However, when present at high tissue concentration, Mn produces cellular toxicity, including neurotoxicity in brain. Although the mechanisms by which Mn induces neuronal damage are not well defined, Mn neurotoxicity appears to be regulated by a number of factors, including oxidative injury, mitochondrial dysfunction, and neuroinflammation (Milatovic et al. 2007, 2009; Zhang et al. 2013). Occupational Mn exposure results in an imbalance between dopamine (DA) and γ-aminobutyric acid (GABA) in the basal ganglia, eliciting the syndrome called manganism, which shares multiple features with Parkinson’s disease (Huang 2007). The strongest correlation between any type of environmental exposure and increased susceptibility to Parkinsonism is observed in the Mn-exposed population (Gorell et al. 1999; Racette et al. 2001).

Occupational exposure (miners, smelters, welders, and workers in dry-cell battery factories; also inhalation of Mn in aerosols/dusts) accounts for the major source of Mn intoxication in humans. An important but non-occupational source of Mn is methylcyclopentadienyl manganese tricarbonyl (MMT), the antiknock agent in gasoline. Another source is potassium permanganate, a powerful oxidizing agent for purifying drinking water, treating waste water, and as an agricultural fungicidal and bactericidal agent (Huang 2007). Manganism has been observed recently in intravenous methcathinone abusers because this substance is illicitly produced by a potassium permanganate oxidation process (Stepens et al. 2008). Additionally, Mn is a natural component of many foods, particularly of nuts, grains, and tea, and is an essential trace element used by humans in enzymatic processes. There are numerous reports of Mn intoxication related to long-term total parenteral nutrition (Reynolds et al. 1994; Reimund et al. 2000).

The effects of Mn on the adult mammalian brain have been studied for decades in connection with manganism. However, the risk of Mn-induced neurotoxicity during brain development, both pre- and postnatally, has received little attention. Several studies report the aftereffects (e.g., reduced intellectual function) of Mn exposure on children at various developmental stages (Woolf et al. 2002; Wasserman et al. 2006). Mn crosses the placenta to impair embryonic development (Spencer 1999) and produce behavioral abnormalities through childhood and into adulthood (Kwieciński and Nowak 2009; Nowak et al. 2010, 2011; Brus et al. 2012; Szkilnik et al. 2014). It is evident that Mn in excess is toxic to human embryos and fetuses (Colomina et al. 1996). The progressive and latent nature of some neurodegenerative disorders (e.g., Parkinson’s disease) suggest that the triggering event for these disorders occurs much sooner than the appearance of visible symptoms. In humans, only after the loss of about 80 % of pars compacta substantia nigra DA neurons do symptoms (e.g., Parkinsonian tremor) arise. Therefore, it is important to identify possible environmental trigger(s), to pinpoint the period during which such factors pose the greatest risk, and to determine the mechanism(s) involved.

The current study was conducted in order to assess the effects of paired gestational Mn exposure with overt dopaminergic neurotoxicity (i.e., 6-hydroxydopamine, 6-OHDA) on hydroxyl radical (HO^•^) production in rat brain. A better understanding of the causes of HO^•^ production in brain and the effects of on neurodegenerative processes are especially important because the brain is at high risk for oxidative injury because of (1) high levels of oxygen consumption combined with (2) low catalase, superoxide dismutase, and glutathione peroxidase activity, (3) high iron concentration, and (4) elevated polysaturated fatty acid content of neuronal membranes (Halliwell 2006).

## Materials and Methods

### Animals and Treatment

Pregnant Wistar rats, 220–250 g, were used in this study. All rats were housed in a well-ventilated room at 22 ± 2 °C with a 12 h light/dark cycle. From the first day of pregnancy (as determined by the presence of vaginal plugs), rats were singly housed with free access to pelleted food (Altromin-1324, Lage, Germany) and tap water containing 10,000 ppm manganese chloride (MnCl_2_·4H_2_O; POCh Ltd., Gliwice, Poland). Control rats received water without Mn. Fluid consumption by each dam was monitored daily. Three days after birth, rats from both groups (control and Mn-exposed) were pretreated with desipramine hydrochloride (20 mg/kg, i.p., base; 1 h; Sigma – a norepinephrine transport inhibitor) and pargyline hydrochloride (50 mg/kg, i.p., salt form; 0.5 h; Sigma) to protect noradrenergic neurons. Rats were then given bilateral intracerebroventricular (i.c.v.) injections with 6-OHDA hydrobromide at one of three doses (15, 30, or 67 µg, salt form, on each side). Control animals received injections of vehicle, consisting of 0.85 % saline and 0.1 % ascorbic acid. This procedure has been described in detail elsewhere (Gong et al. 1993). Rats were weaned on postnatal day 21 (P21), at which time Mn was discontinued, and male offspring was group housed until experimentation. This experiment was approved by the local Bioethical Committee for Animals, Medical University of Silesia (permission no 19/06 issued on 01.03.2006). All procedures, reviewed and approved by the Institutional Animal Care Committee, are in accordance with the principles and guidelines described in the NIH booklet *Care and Use of Laboratory Animals*.

### Manganese Assay

Mn was assayed in control and Mn-exposed dams immediately following parturition. Rats were sacrificed; and the frontal cortex, neostriatum, and hippocampus were dissected. The kidney, liver, femoral bone, femoral muscle, and heart muscle also were taken as samples. Mn levels were estimated in both control and Mn-exposed rats at P14 and P56. Approximately, 100 mg of each tissue was dissolved in 1.0 ml of ultra-pure nitric acid (Merck). Next, Mn^2+^ content was assayed using SP-2900 Pye Unicam AA (Cambridge, UK) atomic absorption spectrometer and handled according to the Company’s brochure (Whiteside 1976). Results were presented in μg/g wet tissue.

### Assessment of 2.3- and 2.5-dihydroxybenzoic Acid and Salicylic Acid in Dissected Tissue

The indirect method of Giovanni et al. (1995) was applied for assessment of HO^•^ generation. This method is based on quantitative, chromatographic measurement of 2.3-dihydroxybenzoic acid (2.3-DHBA), 2.5-dihydroxybenzoic acid **(**2.5-DHBA), and salicylic acid in the examined part of the brain. Control and Mn-intoxicated rats were injected with salicylic acid (100 mg/kg, i.p.), 30 min before decapitation. In 14-day-old offspring, the right hemisphere was taken for analysis; in 8-week-old rats, the frontal cortex, hippocampus, neostriatum, thalamus, cerebellum, and pons were dissected and frozen on dry ice and stored at −80 °C until analysis. Tissue samples were homogenized for 15–20 s. in ice-cold trichloracetic acid (0.1 M) with 0.05 mM ascorbic acid. After centrifugation (5,000×*g* for 10 min), the supernatants were filtered through 0.2 μm cellulose membranes (Titan MSF Microspin Filters, Scientific Resources Inc., Eatontown, GB) and supernatants were injected onto a DHBA-250, 250 × 4 mm, 5 µm column (ESA, USA). Mobile phase consisted of 50 mM sodium acetate (Merck), 50 mM citric acid (Merck), 25 % methanol (J.T. Backer), and 5 % isopropanol (Merck) adjusted to pH 2.5 with phosphoric acid (Fluka). Flow rate was set at 0.5 ml/min. A guard cell at a potential of +775 mV, an E_1_ electrode at +250 mV and 50 nA/V sensitivity, and an E_2_ electrode at +750 mV and 10 μA/V sensitivity were used for 2.3- and 2.5-DHBA and salicylic acid analysis. A Coulochem data analysis system was used to integrate peak areas. Results obtained from 2.3- and 2.5-DHBA assays are presented in units of nanogram per gram of wet tissue (ng/g), and results from salicylic acid assays are given in μg/g.

### Assessment of 2.3- and 2.5-dihydroxybenzoic Acid Via In Vivo Microdialysis

Cannula implantation for in vivo microdialysis was performed in urethane-anesthetized rats. Rats were first placed in a stereotaxic frame. Diazepam (Polfa; 10 mg/kg, i.p.) and ketamine (Parke-Davis; 80 mg/kg, i.p.) were used to provide anesthesia, while the skin and tissue were retracted to expose the skull overlying the neostriatum. A small hole was drilled to allow implantation of the dialysis probe, with 4 mm active membrane delivered (ID 75 µm, OD 150 µm, Polymicron Technologies, USA) into the right neostriatum (A +0.7, L +3.0, V –7.0), coordinates by Paxinos and Watson (1986). Two stainless steel screws were mounted to the cranium near the probe, and this assembly was fixed in place with dental cement (Duracryl Plus, Spofa, Praha). On the following day, rats were injected with urethane (1.5 g/kg, i.p.), and the free ends of the probe were connected with Teflon tubes and continuously perfused at a flow rate of 2.0 µl/min (Microdialysis pump, Harvard Apparatus Model 22, GB) with artificial cerebrospinal fluid (aCSF), consisting of 145 mM NaCl (Merck), 2.7 mM KCl (Fisher), 1.0 mM MgCl_2_·6H_2_O (Aldrich), 1.2 mM CaCl_2_·2H_2_O (Aldrich), and 2.0 mM Na_2_HPO_4_ (Fluka), adjusted to pH 7.4 with phosphoric acid (Fluka). Samples were collected every 22 min and injected directly onto a DHBA-250, 250 × 4 mm, 5 µm column (ESA, USA) with the mobile phase contents the same as for DHBA tissue analysis. After 3 periods of sampling, aCSF was replaced with aCSF containing 5 mM salicylic acid. A guard cell at a potential of +775 mV, an E_1_ electrode at +250 mV with 50 nA/V sensitivity, and an E_2_ electrode at +750 mV with 10 μA/V sensitivity were used for 2.3- and 2.5-DHBA analysis. The study was continued until 220 min. Final results of the 2.3- and 2.5-DHBA were expressed in picograms (pg) in 20 μl capacity of loop samples (Takeda et al. 1990; Nowak et al. 2008, 2010).

### Determination of Superoxide Dismutase (SOD) Activity

The method of Oyanagui (1984) was used to measure the activity of SOD. In this method, xanthine oxidase produces superoxide anions which react with hydroxylamine, forming nitric ions. These ions react with naphthalene diamine and sulfanilic acid, generating a colored product. The concentration of this product is proportional to the amount of generated superoxide anions and negatively proportional to the activity of SOD. Absorbance was measured using an automated Perkin Elmer analyzer at wavelength of 550 nm. The enzymatic activity of SOD was expressed in nitric units. The isoenzymes of SOD, such as Mn-SOD and CuZn-SOD, were also assessed, using KCN as the inhibitor of the CuZn-SOD activity. The activity of SOD is equal to 1 nitric unit (NU) when it inhibits nitric ion production by 50 %. Activities of SOD were expressed in NU/mg protein.

### Determination of Glutathione Reductase (GR) Activity

The activity of GR was measured according to Richterich (1971) using an automated Perkin Elmer analyzer. The activity was expressed as µmoles of NADPH utilized per minute per g (U/g protein).

### Determination of Glutathione S-transferase (GST) Activity

The activity of GST was measured according to the kinetic method of Habig and Jakoby (1981) using an automated Perkin Elmer analyzer. The activity of GST was expressed as µmoles of thioether produced per minute per g protein (U/g protein).

### Determination of Glutathione Peroxidase (GPx) Activity

GPx activity was measured by the kinetic method of Paglia and Valentine (1967). In this method, GPx catalyzes the reaction between reduced glutathione (GSH) and t-butyl hyperoxide. The resulting oxidized glutathione (GSSG) is then converted back to the reduced form (GSH) by a NADPH-dependent glutathione reductase (GR). This reaction results in decreased absorbance at 340 nm which is directly proportional to the GPx activity. The decrease in absorbance was measured by an automated analyzer Perkin Elmer. The activity of GPx was expressed as micromoles of NADPH oxidized per minute per g protein (U/g Hb).

### Determination of Catalase (CAT) Activity

Catalase activity was measured by the method of Johansson and Borg (1988) using an automated Perkin Elmer analyzer. This method is based on the reaction of the enzyme with methanol in the presence of optimal concentrations of hydrogen peroxide. Generated formaldehyde is measured spectrophotometrically at 550 nm as a purple dye. The activity of CAT was expressed as U/g protein.

## Data Analysis

For the Mn assay, Student’s *t* test was used to estimate the significance of the differences between groups. For the remainder of data, two-way analysis of variance (ANOVA) and the post-ANOVA test of Neuman–Keuls were used to test the differences between groups for significance. A *p* value of 0.05 or less was used to indicate a significant difference.

## Results

### Manganese Assay

**Fig. 1:**
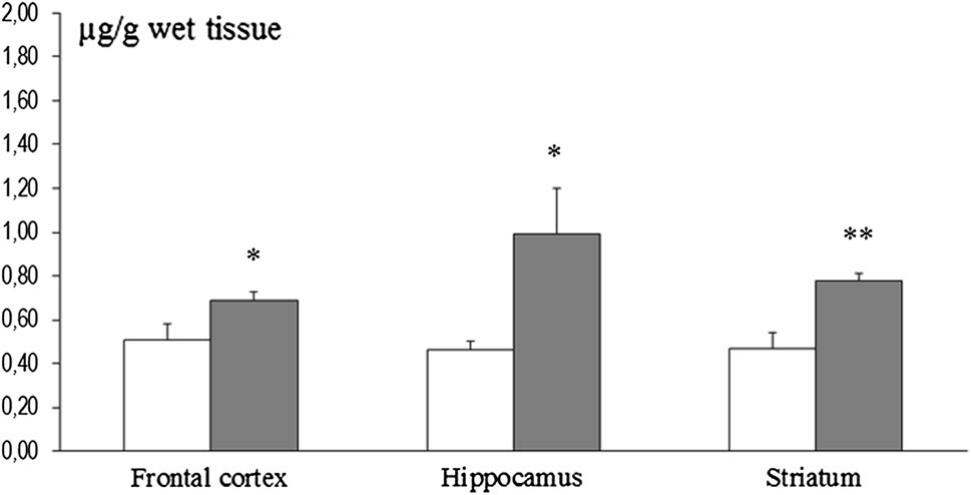
Brain region-specific manganese content in rat dams exposed to manganese (10,000 ppm) during pregnancy and for the first 21 days after birthing (*n* = 5–6). Legend: *open square* control *filled light gray square* Mn (10,000 ppm) * *p* < 0.05; ** *p* < 0.001

**Fig. 2:**
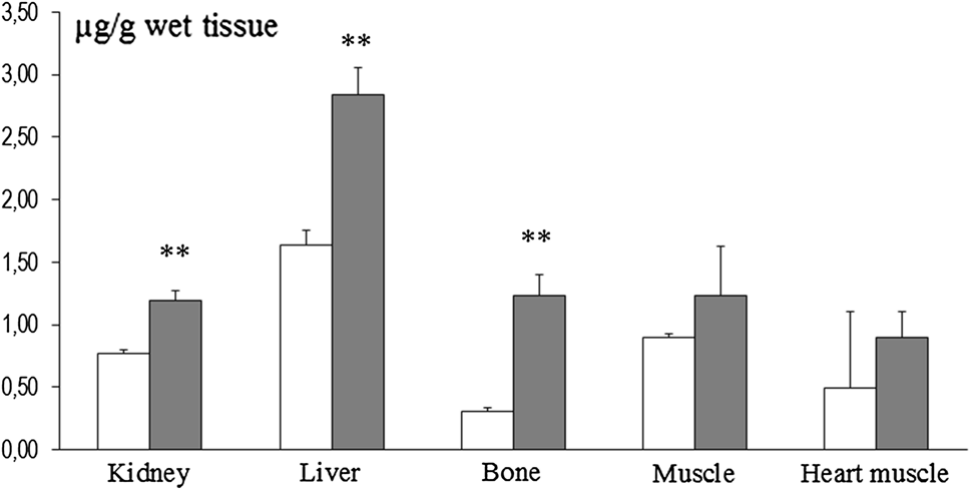
Tissue- and organ-specific manganese content in rats dams exposed to manganese (10,000 ppm) during pregnancy and for the first 21 days after birthing (*n* = 5–6). Legend is the same as in Fig. 1

**Fig. 3:**
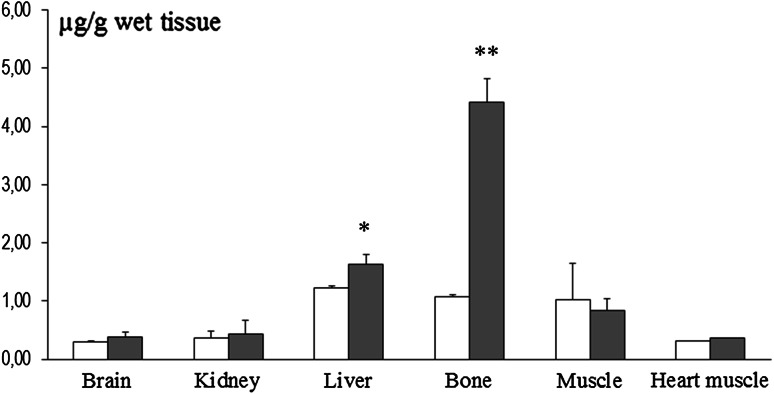
Manganese content in the brain and other tissues and organs in P14 rats pups exposed to this metal (10,000 ppm) during pre- and perinatal development (*n* = 5–6). Legend is the same as in Fig. 1. * *p* < 0.05; ** *p* < 0.01

**Fig. 4:**
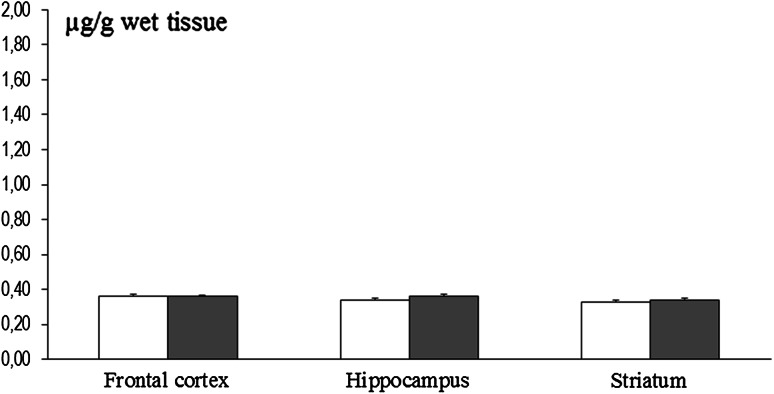
Brain region-specific manganese content in 8-week-old rats exposed to this metal (10,000 ppm) during pre- and perinatal development (*n* = 6). Legend is the same as in Fig. 1

**Fig. 5:**
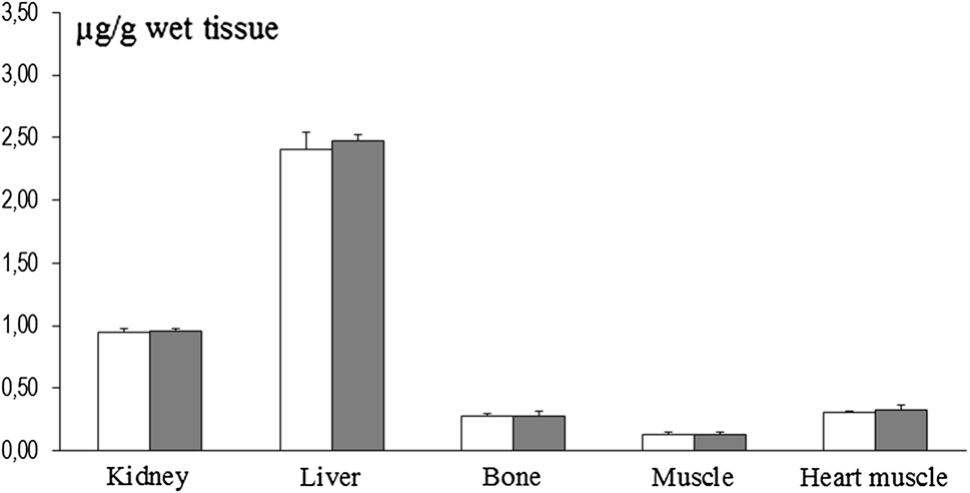
Tissue- and organ-specific manganese content in 8-week-old rats exposed to this metal (10,000 ppm) during pre- and perinatal development (*n* = 6). Legend is the same as in Fig. 1

Mn content in the frontal cortex, hippocampus, and neostriatum was significantly elevated in dams exposed to this metal during pregnancy and until weaning of pups, as compared to control rats [Fig. 1]. Additionally, significant elevations in Mn concentration were observed in the kidney, liver, and bone. Conversely, the Mn content in the femoral muscle and heart muscle was in the range of the respective control values [Fig. 2]. The primary organs showing accumulation of Mn in P14 pups from Mn-exposed mothers were the femoral bones and liver. Interestingly, there were no significant changes in Mn concentration in the brain, kidney, femoral bone, or heart muscle of P14 Mn-exposed pups [Fig. 3]. In 8-week-old rats, the concentration of Mn in all examined tissues was comparable between control and Mn-exposed rats [Figs. 4, 5].

### Assessment of 2.3- and 2.5-dihydroxybenzoic Acid and Salicylic Acid in Tissue Samples

The generation of HO^•^ was estimated from the production of 2.3- and 2.5-DHBA after systemic (i.p.) salicylic acid administration. In 14-day-old rats, 6-OHDA treatment (30, 60, or 134 µg) at P3 was associated with significantly elevated 2.3- and 2.5-DHBA levels in the brain in comparison to control. Similar effects were also observed in P14 Mn-exposed rats injected at P3 with 6-OHDA (Mn vs. Mn + 6-OHDA). Mn exposure alone resulted in an increase in DHBA (*p* < 0.05) in comparison to control animals. Furthermore, in Mn-exposed rats pretreated with 6-OHDA, a significant elevation in DHBA was observed when compared to the group receiving Mn alone. Salicylic acid levels did not differ among the groups of 14-day-old rats [Table 1].

**Table 1:** Concentrations of 2.3- and 2.5-DHBA and salicylic acid in the brains of newborn rats exposed to manganese (10,000 ppm) during pre- and perinatal development (*n* = 6)

14-days newborn rats		Control				Mn			
		0.9 %NaCl	6-OHDA (30 μg)	6-OHDA (60 μg)	6-OHDA (134 μg)	0.9 %NaCl	6-OHDA (30 μg)	6-OHDA (60 μg)	6-OHDA (134 μg)
Right hemisphere	2.3-DHBA (ng/g)	6.25 ± 0.73	9.05* ± 1.13	10.52** ± 0.91	11.25* ± 1.57	8.70# ± 0.69	12.17* ± 1.26	10.03 ± 0.91	11.36* ± 1.17
	2.5-DHBA (ng/g)	20.28 ±2.72	29.13* ± 3.24	30.21* ± 2.87	30.65* ± 2.74	28.39# ± 1.23	38.13* ± 4.22	32.93* ± 2.74	41.01* ± 5.23
	SAL (μg/g)	22.75 ± 2.50	22.83 ± 2.44	25.71 ± 0.75	23.83 ± 3.63	23.23 ± 1.97	24.09 ± 1.50	26.23 ± 1.99	25.70 ± 2.30

* *p* < 0.05

* *p* < 0.001 control versus control + 6-OHDA or manganese versus manganese + 6-OHDA

# *p* < 0.05 control versus manganese

In prefrontal cortex of 8-week-old rats, 2.3-DHBA was elevated in control rats (i.e., no Mn exposure) treated at P3 with 6-OHDA at doses of 60 and 134 µg, whereas 2.5-DHBA levels were elevated in all 6-OHDA groups (30, 60, and 134 µg). Mn exposure alone resulted in a significant increase in 2.3- and 2.5-DHBA contents in prefrontal cortex versus rats without Mn exposure. 6-OHDA treatment did not enhance effects of Mn on 2.3- and 2.5-DHBA contents [Table 2]. Similar effects relating to 6-OHDA treatment and Mn exposure were observed in the hippocampus [Table 3] and thalamus [Table 4]. In the brain stem, 2.3-DHBA contents were unaltered at 8 weeks after 6-OHDA and/or Mn exposure. However, 2.5-DHBA was elevated in control rats (i.e., no Mn exposure) treated at P3 with 6-OHDA (30 and 60 µg) and in rats exposed perinatally to Mn and not treated at P3 with 6-OHDA [Table 5]. No significant changes in 2.3- and 2.5-DHBA were observed in the cerebellum [Table 6]. In the neostriatum, 6-OHDA treatment (60 or 134 µg) at P3 significantly elevated 2.3- and 2.5-DHBA levels at 8 weeks. Similar effects were also observed in rats exposed perinatally to Mn. Moreover, in Mn-exposed rats that were treated at P3 with 6-OHDA, 2,3- and 2,5-DHBA levels were further elevated. Salicylic acid levels did not differ among the groups of 8-week-old rats [Table 7].

**Table 2:** Concentrations of 2.3- and 2.5-DHBA and salicylic acid in the prefrontal cortex of adult rats exposed to manganese (10,000 ppm) during pre- and perinatal development (*n* = 5–7)

Part of the brain		Control				Mn			
		0.9 %NaCl	6-OHDA (30 μg)	6-OHDA (60 μg)	6-OHDA (134 μg)	0.9 %NaCl	6-OHDA (30 μg)	6-OHDA (60 μg)	6-OHDA (134 μg)
Prefrontal cortex	2.3-DHBA (ng/g)	3.25 ± 0.45	4.40 ± 0.56	4.66* ± 0.35	4.69* ± 0.49	4.90# ± 0.59	4.58 ± 0.61	4.39 ± 0.63	5.62 ± 0.76
	2.5-DHBA (ng/g)	17.68 ± 0.81	25.88* ± 2.60	28.48** ± 2.98	38.15** ± 5.15	26.32## ± 2.57	21.28 ± 2.38	26.42 ± 4.39	24.92 ± 1.19
	SAL (μg/g)	11.05 ± 0.64	12.78 ± 1.73	11.23 ± 0.40	14.18 ± 1.55	13.44 ± 1.53	10.75 ± 0.88	10.94 ± 1.52	13.81 ± 1.36

* *p* < 0.05

** *p* < 0.005 control versus control + 6-OHDA or manganese versus manganese + 6-OHDA

# *p* < 0.05

## *p* < 0.005 control versus manganese

**Table 3:** Concentrations of 2.3- and 2.5-DHBA and salicylic acid in the hippocampus of adult rats exposed to manganese (10,000 ppm) during pre- and perinatal development (*n* = 5–7)

Part of the brain		Control					Mn		
		0.9 %NaCl	6-OHDA (30 μg)	6-OHDA (60 μg)	6-OHDA (134 μg)	0.9 %NaCl	6-OHDA (30 μg)	6-OHDA (60 μg)	6-OHDA (134 μg)
Hippocampus	2.3-DHBA (ng/g)	3.55 ± 0.47	5.19* ± 0.61	4.91 ± 0.60	5.84* ± 0.77	4.73# ± 0.33	4.62 ± 0.45	3.78 ± 0.33	5.87 ± 0.89
	2.5-DHBA (ng/g)	16.58 ± 1.29	21.32 ± 2.48	21.76* ± 1.59	30.40* ± 6.70	21.43# ± 1.58	22.95 ± 1.67	25.23 ± 2.35	25.81 ± 1.90
	SAL (μg/g)	11.70 ± 1.78	13.16 ± 1.21	12.29 ± 1.19	13.24 ± 2.68	12.31 ± 0.40	13.81 ± 0.88	11.31 ± 1.18	14.13 ± 1.07

* *p* < 0.05 control versus control + 6-OHDA or manganese versus manganese +6-OHDA

# *p* < 0.05 control versus manganese

**Table 4:** Concentrations of 2.3- and 2.5-DHBA and salicylic acid in the thalamus of adult rats exposed to manganese (10,000 ppm) during pre- and perinatal development (*n* = 5–7)

Part of the brain		Control				Mn			
		0.9 %NaCl	6-OHDA (30 μg)	6-OHDA (60 μg)	6-OHDA (134 μg)	0.9 %NaCl	6-OHDA (30 μg)	6-OHDA (60 μg)	6-OHDA (134 μg)
Striatum	2.3-DHBA(ng/g)	317 ± 0.28	3.29 ± 1.48	4.59* ± 0.38	4.18* ± 0.45	4.39 ± 0.37	6.04* ± 0.50	5.97 ± 0.50	6.28* ± 0.81
	2.5-DHBA (ng/g)	15.48 ± 1.24	19.44 ± 1.97	23.08* ± 3.15	27.25** ± 2.16	19.95# ± 1.62	24.10* ± 1.76	25.36* ± 2.33	26.52* ± 2.18
	SAL (μg/g)	10.02 ± 0.59	11.01 ± 1.15	10.18 ± 0.73	11.42 ± 1.52	12.29 ± 0.57	11.80 ± 0.57	10.21 ± 0.58	12.35 ± 1.52

* *p* < 0.05

** *p* < 0.005 control versus control + 6-OHDA or manganese versus manganese +6-OHDA

# *p* < 0.05 control versus manganese

**Table 5:** Concentrations of 2.3- and 2.5-DHBA and salicylic acid in the brain stem of adult rats exposed to manganese (10,000 ppm) during pre- and perinatal development (*n* = 5–7)

Part of the brain		Control				Mn			
		0.9 %NaCl	6-OHDA (30 μg)	6-OHDA (60 μg)	6-OHDA (134 μg)	0.9 %NaCl	6-OHDA (30 μg)	6-OHDA (60 μg)	6-OHDA (134 μg)
Thalamus and hypothalamus	2.3-DHBA (ng/g)	7.60 ± 0.82	15.64** ± 1.67	12.92* ± 1.56	8.33 ± 1.05	21.53# ± 5.46	19.84 ± 1.63	19.24 ± 1.47	20.76 ± 5.77
	2.5-DHBA (ng/g)	37.21 ± 4.01	44.81 ± 4.89	52.24 ± 9.52	58.63** ± 5.16	63.06# ± 8.32	59.33 ± 4.62	54.64 ± 3.15	62.15 ± 15.39
	SAL (μg/g)	14.03 ± 1.44	16.64 ± 1.93	18.09 ± 2.15	14.24 ± 1.35	16.14 ± 1.69	16.94 ± 1.66	14.40 ± 1.71	15.02 ± 1.56

* *p* < 0.05 control versus control + 6-OHDA or manganese versus manganese +6-OHDA

# *p* < 0.05 control versus manganese

**Table 6:** Concentrations of 2.3- and 2.5-DHBA and salicylic acid in the cerebellum of adult rats exposed to manganese (10,000 ppm) during pre- and perinatal development (*n* = 5–7)

Part of the brain		Control				Mn			
		0.9 %NaCl	6-OHDA (30 fig)	6-OHDA (60 μg)	6-OHDA (134 μg)	0.9 %NaCl	6-OHDA (30 μg)	6-OHDA (60 μg)	6-OHDA (134 μg)
Cerebellum	2.3-DHBA(ng/g)	4.86 ± 0.86	7.99 ± 1.48	6.91 ± 1.43	6.94 ± 1.08	6.99 ± 0.71	7.13 ± 0.61	8.16 ± 1.02	8.23 ± 1.16
	2.5-DHBA (ng/g)	40.10 ± 7.15	52.56 ± 9.32	53.59 ± 6.95	54.09 ± 5.34	45.71 ± 3.47	52.02 ± 6.65	54.10 ± 11.66	43.27 ± 4.09
	SAL (μg/g)	11.83 ± 1.34	12.95 ± 1.33	11.70 ± 1.26	11.25 ± 0.47	11.68 ± 0.78	12.54 ± 1.00	11.60 ± 1.40	14.36 ± 1.67

**Table 7:** Concentrations of 2.3- and 2.5-DHBA and salicylic acid in the neostriatum of adult rats exposed to manganese (10,000 ppm) during pre- and perinatal development (*n* = 5–7)

Part of the brain		Control				Mn			
		0.9 %NaCl	6-OHDA (30 μg)	6-OHDA (60 μg)	6-OHDA (134 μg)	0.9 %NaCl	6-OHDA (30 μg)	6-OHDA (60 μg)	6-OHDA (134 μg)
Brain stem	2.3-DHBA (ng/g)	4.23 ± 1.19	5.87 ± 0.98	5.55 ± 0.75	5.60 ± 1.42	5.02 ± 0,59	4.64 ± 0.59	4.61 ± 0.61	4.81 ± 0.75
	2.5-DHBA (ng/g)	26.00 ± 2.51	42.43* ± 6.27	35.41* ± 3.26	31.41 ± 4.13	37.41# ± 4.61	36.46 ± 3.64	32.50 ± 3.04	24.99 ± 2.00
	SAL (μg/g)	11.31 ± 1.14	11.94 ± 1.58	10.12 ± 1.07	9.30 ± 0.30	10.23 ± 0.80	10.06 ± 0.95	9.69 ± 0.85	10.60 ± 0.63

* *p* < 0.05

** *p* < 0.005 control versus control + 6-OHDA or manganese versus manganese +6-OHDA

# *p* < 0.05 control versus manganese

### Assessment of 2.3- and 2.5-dihydroxybenzoic acid Microdialysate Contents

**Fig. 6:**
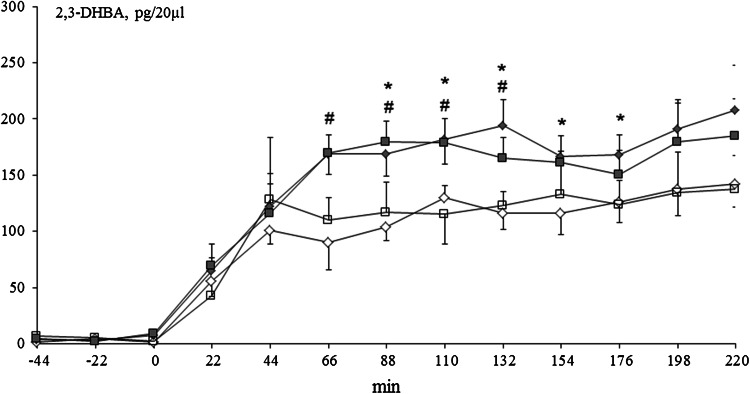
Striatal microdialysate 2.3-DHBA concentrations in control and manganese-exposed (10,000 ppm) rats lesioned with 6-OHDA (*n* = 5–7). Legend: *open diamond* control *open square* manganese *filled diamond* control + 6-OHDA (134 µg) *filled square* manganese + 6-OHDA (134 µg) * *p* < 0.05 control versus control + 6-OHDA (134 µg) # *p* < 0.05 manganese versus manganese + 6-OHDA (134 µg)

**Fig. 7:**
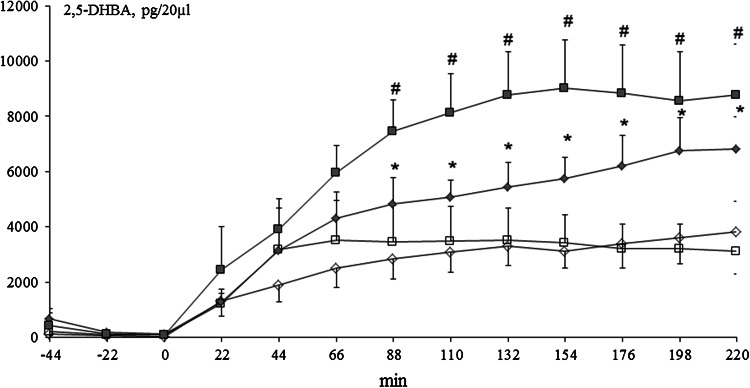
Striatal microdialysate 2.5-DHBA concentrations in control and manganese-exposed (10,000 ppm) rats lesioned with 6-OHDA (*n* = 5–7). Legend is the same as in Fig. 6

After switching aCSF to aCSF with 5 mM salicylic acid (point “0” on the graph), 2.3- and 2.5-DHBA appeared in the microdialysates of the neostriatum. The formation of 2.3-DHBA stabilized at 44 min in control and Mn-exposed rats and reached a concentration of ~100 pg/20 µl in both groups. In control rats treated at P3 with 6-OHDA (134 µg) as well as in Mn-exposed rats treated at P3 with 6-OHDA (134 µg), 2.3-DHBA levels stabilized at 66 min and attained a concentration of ~180 pg/20 µl. Significant differences between control and control + 6-OHDA groups were observed at 88, 110, 132, 154, and 176 min (*p* < 0.05), whereas differences were observed between Mn and Mn + 6-OHDA groups at 88, 110, and 132 min of testing [Fig. 6]. With respect to the 2.5-DHBA microdialysate, significant differences between control vs. control + 6-OHDA and Mn vs. Mn + 6-OHDA were noted between 88 and 220 min of the experiment [Fig. 7].

### Assessment of Antioxidant Enzyme Activities

**Fig. 8:**
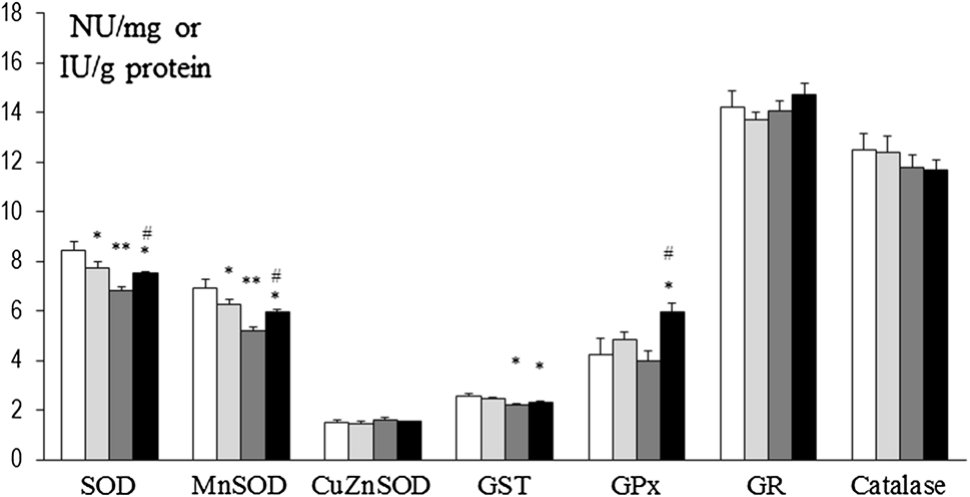
Antioxidant enzymes activity in prefrontal cortex of adult control and manganese-exposed (10,000 ppm) rats lesioned with 6-OHDA (*n* = 8). Legend: *open square* control *filled* *light gray bar* control + 6-OHDA (134 µg) *filled* *dark gray bar* manganese *filled black square* manganese + 6-OHDA (134 µg) * *p* < 0.05; ** *p* < 0.01 control versus 6-OHDA; control versus manganese; control versus manganese + 6-OHDA # *p* < 0.05 manganese versus manganese + 6-OHDA

**Fig. 9:**
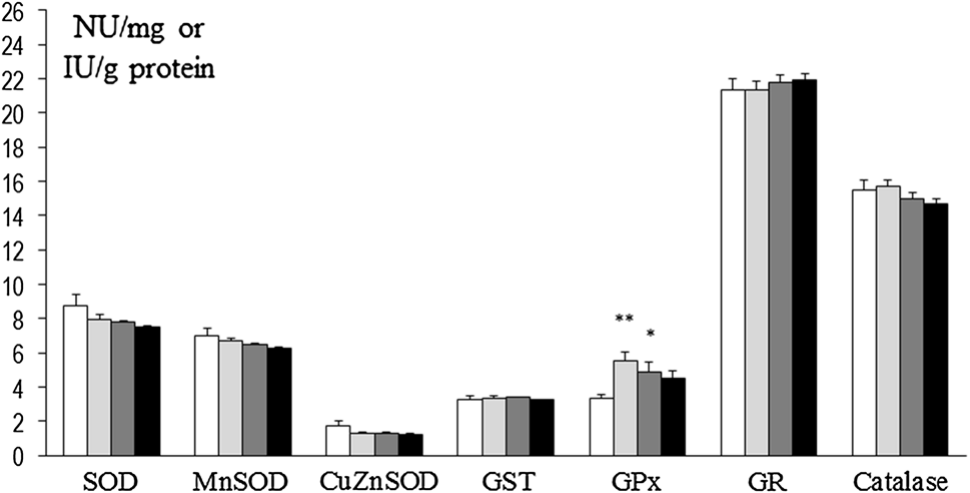
Antioxidant enzymes activity in hippocampus of adult control and manganese-exposed (10,000 ppm) rats lesioned with 6-OHDA (*n* = 8). Legend is the same as in Fig. 8. * *p* < 0.05; ** *p* < 0.01 Control versus 6-OHDA and control versus manganese

**Fig. 10:**
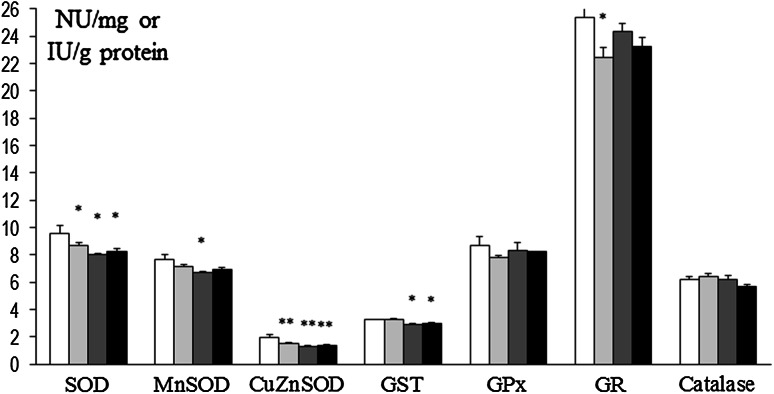
Antioxidant enzymes activity in striatum of adult control and manganese-exposed (10,000 ppm) rats lesioned with 6-OHDA (*n* = 8). Legend is the same as in Fig. 8. * *p* < 0.05; ** *p* < 0.01 control versus 6-OHDA; control versus manganese; control versus manganese + 6-OHDA

**Fig. 11:**
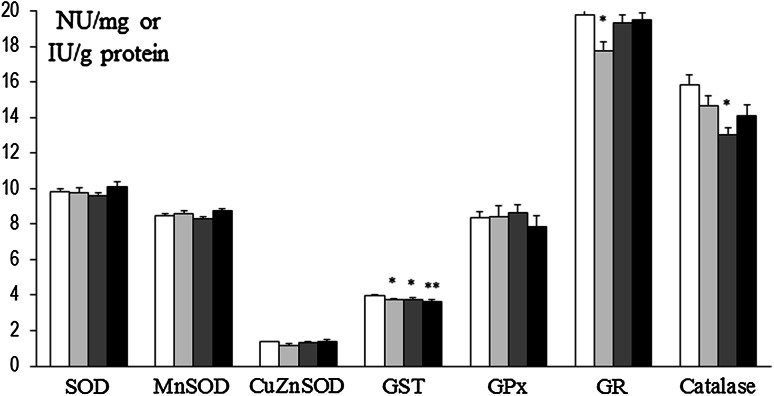
Antioxidant enzymes activity in thalamus and hypothalamus of adult control and manganese-exposed (10,000 ppm) rats lesioned with 6-OHDA (*n* = 8). Legend is the same as in Fig. 8. * *p* < 0.05; ** *p* < 0.01 control versus 6-OHDA; control versus manganese; control versus manganese + 6-OHDA

**Fig. 12:**
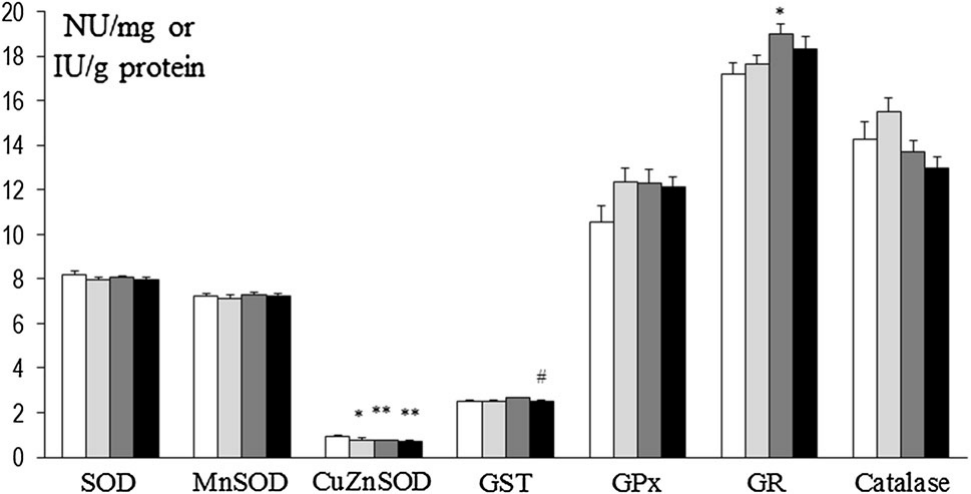
Antioxidant enzymes activity in cerebellum of adult control and manganese-exposed (10,000 ppm) rats lesioned with 6-OHDA (*n* = 8). Legend is the same as in Fig. 8. * *p* < 0.05; ** *p* < 0.01 control versus 6-OHDA; control versus manganese; control versus manganese + 6-OHDA #*p* < 0.05 manganese versus manganese + 6-OHDA

**Fig. 13:**
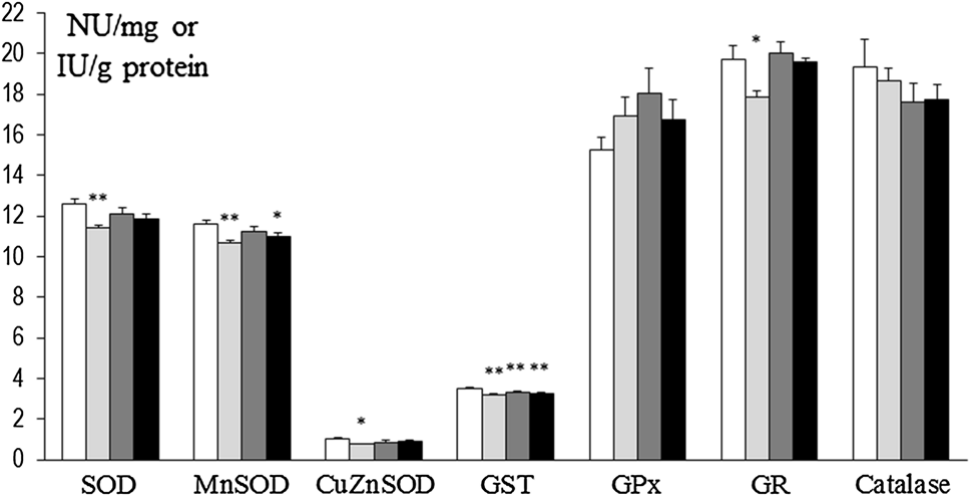
Antioxidant enzymes activity in brain stem of adult control and manganese-exposed (10,000 ppm) rats lesioned with 6-OHDA (*n* = 8). Legend is the same as in Fig. 8. * *p* < 0.05; ** *p* < 0.01 control versus 6-OHDA; control versus manganese; control versus manganese + 6-OHDA

In prefrontal cortex at 8 weeks, SOD and Mn-SOD activities were decreased in rats that had been treated at P3 with 6-OHDA (134 µg) as well as in Mn and Mn + 6-OHDA groups versus control. Also, concurrently in Mn + 6-OHDA, rats activities of these enzymes were significantly elevated versus respective controls (Mn alone exposed rats). GST was decreased in Mn and Mn + 6-OHDA groups versus control, while GPx was significantly elevated in Mn + 6-OHDA rats (versus control and Mn groups). Activities of CuZn-SOD, GR, and catalase were unchanged [Fig. 8]. In hippocampus, only GPx activity was changed (elevated in 6-OHDA and Mn groups); activity of other enzymes was unchanged [Fig. 9]. In striatum SOD and CuZn-SOD, activities were decreased in 6-OHDA, Mn, and Mn + 6-OHDA groups. Significant decreases in Mn-SOD (only in Mn alone exposed rats), GST (Mn and Mn + 6-OHDA), and GR (6-OHDA) activities [Fig. 10]. In thalamus and hypothalamus, 6-OHDA (134 µg) produced significant decreases in the activities of GST and GR, Mn in GST and catalase, and Mn + 6-OHDA in GST activity [Fig. 11]. In cerebellum, CuZn-SOD activity was decreased in 6-OHDA-lesioned rats as well as in Mn alone exposed rats and Mn + 6-OHDA rats. Also, GST activity was decreased in the Mn + 6-OHDA versus control (Mn alone). Conversely, GR activity was elevated in Mn-exposed rats as compared to controls [Fig. 12]. In brain stem, 6-OHDA (134 µg) produced a decrease in the activities of SOD, Mn-SOD, CuZn-SOD, and GR; also a reduction in Mn-SOD activity was found in the Mn + 6-OHDA group. Furthermore, GST was decreased in the 6-OHDA group, Mn and Mn + 6-OHDA rats versus controls [Fig. 13].

## Discussion

In the present study, we demonstrated that Mn content in the brain (frontal cortex, hippocampus, and neostriatum), kidney, liver, and bone was significantly elevated in rat dams exposed to this metal during pregnancy. Conversely, in neonates (P14) whose mothers were exposed to Mn during the prenatal and perinatal periods, Mn primarily accumulated in the femoral bone and liver. In P14 pups, no significant differences in Mn content in other tissues (brain, kidney, femoral muscle, and heart) were noted in comparison to control. Interestingly, Mn accumulation in the femoral bone and liver was not observed in 8-week-old Mn-exposed rats. This is in agreement with other reports indicating that Mn can be transiently deposited in various rat tissues (mostly in the liver), both after parenteral and oral administration (Roels et al. 1997). Dorman et al. (2005) showed that newborn rats exposed to Mn via aerosol inhalation during lactation had a 2–3-fold increase in Mn content in brain and in other tissues (stomach, blood, liver, and skull cap); however, following cessation of Mn administration, tissue Mn concentrations returned to control values by P45 in all offspring tissues. Others have demonstrated that the level of Mn in perinatally exposed pups was 6–8 times higher than in controls. When Mn intoxication was discontinued, the Mn contents decreased nearly to control levels (Kostial et al. 2005). The data cited above largely corroborate the present findings. It is notable that Siddappa et al. (2002) and Garcia et al. (2006) observed that the expressions of proteins involved in Mn transport, i.e., divalent metal transporter-1 (DMT-1) or transferrin receptors in the central nervous system (cerebral cortex, hippocampus, and neostriatum), appear in rats at P5. This may, at least in part, explain the results of our study concerning the lack of accumulation of this metal in the brains of neonates (14-day-old rats). These studies, as well as our own observations, indicate that Mn crosses the placenta. Although, in contrast to the other heavy metals (cadmium, lead) (Brus et al. 1995; Nowak et al. 2006; Szczerbak et al. 2007), Mn is not deposited in the tissue for long periods of time. Following cessation of Mn exposure, Mn is very quickly and efficiently eliminated by the body.

To determine the possible role of HO^•^ generation in Mn-induced neurotoxicity, we estimated the content of 2.3- and 2.5-DHBA (spin trap products of salicylate; HO^•^ provides an index of in vivo reactive oxygen species generation). We demonstrated that DA denervation resulted in enhancement of HO^•^ formation in the brains of newborn rats. Similar data were obtained from adult animals (8-week-old rats lesioned with 6-OHDA at a dose of 30, 60, or 134 µg at P3). In the current study, 2.3-DHBA and 2.5-DHBA contents were significantly increased in the frontal cortex, hippocampus, neostriatum, thalamus with hypothalamus, and pons of 6-OHDA-lesioned rats. Additionally, there was no association between the extent of DA denervation and increased HO^•^ formation. These data are in agreement with the results published by Kostrzewa et al. (2000) who found that in the DA-denervated neostriatum (6-OHDA, 134 µg), 2.3-DHBA was increased more than 4-fold, and 2.5-DHBA was increased 2.5-fold in comparison to fully DA-innervated rats.

An important novelty of our work is the demonstration that HO^•^ overproduction was observed not only in rats with profound dopaminergic system damage (6-OHDA, 134 μg), but also in animals with intermediate (6-OHDA, 60 μg) and minor (6-OHDA, 30 μg) DA depletion. This indicates that even modest injury to the dopaminergic system acts as a “trigger mechanism,” initiating a cascade of adverse signaling events that lead to a protracted increase in HO^•^ generation. Concurrently, we showed that if this process is launched in early postnatal life (14-day-old rats), then it persists throughout adult life. One hypothesis that could explain this phenomenon is that 6-OHDA administered to laboratory animals induces up-regulation of DMT-1 localized on DA neurons, with subsequent increases in iron overload—a possible substrate for HO^•^ production (Fenton reaction) (Song et al. 2007; Jiang et al. 2010). Furthermore, others found reduced expression of ferroportin 1 and hephaestin in the substantia nigra of 6-OHDA-lesioned rats. These two iron export proteins are involved in removal of this metal from neurons in the substantia nigra (Wang et al. 2007). This hypothesis could also serve as an explanation for the absence of changes in DHBA in the cerebellum, since the cerebellum has sparse dopaminergic innervation, and abnormal iron homeostasis seems to be of marginal relevance to this part of the brain.

In the present work, we also showed that perinatal Mn exposure increases the generation of HO^•^ in the brains of newborn rats; in the frontal cortex, hippocampus, thalamus with hypothalamus, and partly in the pons at 8-weeks. Moreover, 6-OHDA-induced DA denervation enhanced this effect in the neostriatum at P14 and through 8 weeks.

Because of the latter effect, it was of interest to assess neostriatal extraneuronal HO^•^ levels. We found that microdialysate levels of both 2.3- and 2.5-DHBA were significantly elevated in DA-denervated neostriatum. These results were counter to our expectation because the extraneuronal compartment is effectively protected by various antioxidants (e.g., ascorbic acid, uric acid, etc.) prior to HO^•^ formation. Additionally, endogenous melatonin may play an active role in maintaining oxidative homeostasis in the extracellular compartment of the neostriatum (Rocchitta et al. 2005). Extracellular ascorbic acid concentrations in the neostriatum range between 350 and 500 µM, similarly to uric acid; and increases by ~50 % following an increase in evoked DA release (e.g., after systemic amphetamine injection) (Miele and Fillenz 1996).

Interestingly, no differences were found in microdialysate contents of 2.3- and 2.5-DHBA in the neostriatum between Mn-exposed and control rats. These results are counter to findings on isolated neostriatal tissues. However, in isolated neostriatal tissues, the measurement of the intensity in HO^•^ generation reflects an intracellular compartment, which represents more than 90 % of its weight, as well as the extraneuronal compartment. Furthermore, extracellular HO^•^ is dwarfed by the intraneuronal environment wherein the majority of metabolic processes occur (mitochondria, lysosomes, etc.). These considerations find confirmation in the work by Milatovic et al. (2007, 2009) who showed that Mn induces neuronal damage through oxidative injury and mitochondrial dysfunction (intracellular compartment). Conversely, it is known that DA is a source of free radicals, which are formed during its enzymatic metabolism (monoamine oxidase), or by non-enzymatic autoxidation that leads to production of highly reactive DA quinones and DA semiquinones (Halliwell 2006). Under conditions of a marked DA deficit, glutamate could initiate free radicals formation through stimulation of intracellular signaling, in the process termed excitotoxicity. This hypothesis is supported by the study of Golembiowska and Dziubina (2012) who showed that a marked increase in striatal extracellular glutamate level in rats with 6-OHDA-induced DA depletion could account for enhanced extraneuronal formation of hydroxyl radicals. Conversely, restoration of the striatal DA-glutamate balance suppressed 6-OHDA-induced overproduction of hydroxyl radical. In our recently published study in which the same experimental model was engaged (as in the present work), we found no difference in extraneuronal DA in control versus Mn-exposed rats (Szkilnik et al. 2014). Under the assumption that Mn exposure minimally affected the extraneuronal milieu (i.e., DA release), we cannot expect overt changes in extraneuronal hydroxyl radical formation.

DHBA studies coincide with antioxidant enzyme activity deterioration that we found in the brains of rats lesioned with 6-OHDA and/or intoxicated with Mn. The most prominent impairments were observed in the prefrontal cortex, striatum, and brain stem i.e., significant decrease in activity of SOD isoenzymes and GST was noted in 6-OHDA-, Mn-, and Mn + 6-OHDA groups in comparison to control. In mammals, SODs represent the major antioxidant defense system against superoxide anion (O_2_^•−^). We hypothesize that ineffectiveness of SOD activity may result in accumulation of O_2_^•−^ which, in turn, in the presence of hydrogen peroxide (H_2_O_2_) (Haber–Weiss reaction) brings about enhancement of HO^•^ generation, the effect demonstrated in the current study. Furthermore, from our work, we have also learned that H_2_O_2_ cleavage mechanisms are nearly unaffected (in exception with prefrontal cortex and hippocampus) because negligible changes in catalase and glutathione-associated enzymes activity were found. Our data are in agreement with others who also demonstrated that 6-OHDA treatment resulted in impairment (decrease) in antioxidant enzymes activity in rats and mice (Ahmad et al. 2012; Haleagrahara et al. 2013). Also, Santos et al. (2012) found that in rats injected ip for 4 or 8 days with 25 mg MnCl_2_/kg/day, a significant increase in Mn-SOD protein expression was noted in brains after 4 Mn doses, but the expressions of these proteins were decreased after 8 Mn doses. Chtourou et al. (2010) demonstrated that Mn intoxication (20 mg/ml manganese chloride in drinking water for 30 days) was accompanied by a decrease of enzymatic (SOD, CAT, and GPx) and non-enzymatic (glutathione and ascorbic acid) antioxidants in the rat’s cerebral cortex.

In summary, the current study demonstrates HO^•^ overproduction in brain tissue (prefrontal cortex, hippocampus, thalamus/hypothalamus, striatum) and in striatal in vivo (extraneuronal) microdialysates of adulthood rats, despite lack of a measurable residual of Mn consequent to perinatal Mn exposure (i.e., Mn addition to the drinking water of mother rats during gestation and through the 21 day suckling period). Moreover, HO^•^ elevation was enhanced in perinatal Mn-exposed rats by mild-, moderate-, or extensive-postnatal 6-OHDA lesioning of dopaminergic neurons, which alone resulted in increased tissue- and microdialysate levels of HO^•^. On the basis of this study, it appears that perinatal Mn exposure may represent a life-long risk toward the incidence or severity of neurodegenerative disorders, such as Parkinson’s disease, in humans.
